# Clinical features and high-resolution chest computerized tomography findings of children infected by the B.1.617.2 variant of coronavirus disease 2019

**DOI:** 10.1080/07853890.2022.2114608

**Published:** 2022-08-29

**Authors:** Chuanjun Xu, Mengya Ma, Yongxiang Yi, Changhua Yi, Hui Dai

**Affiliations:** a Department of Radiology, The Second Hospital of Nanjing Nanjing University of Chinese Medicine, Nanjing, P.R. China; b Department of Radiology, The First Affiliated Hospital of Soochow University, Suzhou, P.R. China; c Institute of Medical Imaging, Soochow University, Suzhou, P.R. China; d Nanjing Infectious Disease Center, The Second Hospital of Nanjing, Nanjing University of Chinese Medicine, Nanjing, P.R. China; e Nanjing Infectious Diseases Clinical Medical Center, The Second Hospital of Nanjing, Nanjing University of Chinese Medicine, Nanjing, P.R. China

**Keywords:** B.1.617.2 (Delta) variant, COVID-19, CT, chest

## Abstract

**Purpose:**

To analyse the clinical symptoms, laboratory examinations and chest CT findings of children infected by the B.1.617.2 variant of COVID-19 and to compare the differences between clinical subtypes.

**Methods:**

Fifty-three children (28 males, 25 females; age ranging from 4 months to 17 years) were included with B.1.617.2 variant infection in Nanjing, China, from July 21 to August 12 2021. Clinical data from patients were collected and analysed in groups of mild and common types. Imaging data were divided into three stages for evaluation: early, intermediate and late stages.

**Results:**

In our study, fever (53%), cough (34%) and pharyngeal discomfort (28%) were the main symptoms. There were no differences in clinical symptoms between the mild and common type. The most common laboratory test items outside the normal range were decreased mean corpuscular volume (68%), lymphocyte percentage (64% elevated and 2% decreased) and decreased serum alkaline phosphatase concentration (66%). The differences in haemoglobin and monocyte percentages between the mild and common types were statistically significant (*p* = .037 and .033, respectively). No influencing factor was statistically significant in the regression analysis of both symptoms and clinical subtypes. The main CT findings were ground-glass opacity and consolidation located in the periphery and bilateral multilobed involvement. The mean CT score was 1.6. CT score correlated with packet cell volume, haemoglobin, mean erythrocyte volume, mean platelet volume and platelet distribution width.

**Conclusion:**

The pathogenetic condition of children with B.1.617.2 variant infection is mild. Although there were intergroup differences in some blood cell analyses, T-lymphocyte counts, and comprehensive biochemical indicators, no factors had a significant effect on clinical typing and the presence or absence of symptoms. CT findings and CT scores reflect disease stage and pathological changes and correlate moderately with laboratory tests, making them of good value for disease diagnosis and monitoring.

**Key Messages**
- Paediatric patients infected with B.1.617.2 variant have a milder clinical and imaging presentation than adults and are similar to the prototype infection.
- CT findings and scores which reflect disease stages and pathological changes.
- There is a correlation between chest CT and laboratory tests, which can be useful for the diagnosis and follow-up of the disease.

## Introduction

In 2019, a highly infectious but aetiologically unknown respiratory disease occurred in Wuhan, China. Subsequently, it was confirmed to be caused by a coronavirus named severe acute respiratory syndrome coronavirus 2 (SARS-CoV-2) by the International Committee on Classification of Viruses [1]. Coronavirus Disease 2019 (COVID-19) remains a global public health threat. Numerous SARS-CoV-2 variants have emerged. The SARS-CoV-2 B.1.617 lineage was first reported in India in October 2020. The SARS-CoV-2 B.1.617.2 (Delta) variant became the most prevalent in India from mid-April 2021 [2]. The variant was highly infectious and spread rapidly and has now spread to 78 countries [3–4]. Within one day, seven new locally confirmed cases were reported at Nanjing Lukou Airport, Jiangsu Province [5], followed by a rapid increase in infected patients, with an aetiology conformation of SARS-CoV-2 B.1.617.2 (Delta) variant infection.

Based on previously published literature, the symptoms of acute respiratory infection appeared early in COVID-19 in all age groups. The most common symptoms were fever (43.8% on admission and 88.7% during hospitalization) and cough (67.8%) [6], with some infections rapidly progressing to severe complications such as acute respiratory distress syndrome (ARDS) and acute respiratory failure. Acute respiratory distress syndrome (ARDS) is the rapid onset of noncardiogenic pulmonary oedema, hypoxaemia and the need for mechanical ventilation in hospitalized patients [7]. Subsequent studies focussed on the analysis of clinical features achieved good agreement in their conclusions that COVID-19 had a milder course and better prognosis in the paediatric population than in the adult population [8–10]. In children, the common signs and symptoms include fever, cough and pharyngeal erythema [11]. A total of 69.6% (828/1189) of children had a normal leukocyte count [12]. High-resolution chest computerized tomography (CT) was used as an aid to identify suspected COVID-19 cases, and one review summarized that children with abnormal CT presentation accounted for 2/3 of the overall cases. However, the CT presentation was mild, and the benefits of CT outweighed the radiation risks [13].

The currently prevalent SARS-CoV-2 B.1.617.2 (Delta) variant has been less studied in paediatric patients. In the face of its higher infectious threat, whether there are different features of clinical, laboratory tests and imaging findings remains unknown and urgently needs to be elucidated by researchers. This study retrospectively collected positive paediatric patients for SARS-CoV-2 Delta variant infection. Their clinical manifestations, laboratory test results and high-resolution CT features were described to improve the efficiency of identifying and diagnosing suspicious cases during epidemic prevention. Disease development and diagnosis can be better understood, and better implementation of clinical measures.

## Materials and methods

### Participants

This study was conducted in accordance with the amended Declaration of Helsinki. Independent ethics committees approved the protocol, and written informed consent was obtained from all patients. Inpatients at Nanjing Second Hospital (Tangshan Branch) were included in this study from July 21 to August 12 2021. Inclusion criteria were as follows: (1) All cases followed the Diagnosis and Treatment Protocols of Coronavirus Disease 2019 (Trial Version 8 Revised) established by the National Health Commission of the People's Republic of China [14]; (2) Viral gene sequencing showed high homology of pharyngeal swab samples with SARS-CoV-2 B.1.617.2 (Delta) variant; (3) Age less than 18 years. The exclusion criteria were as follows: (1) recent pulmonary infections attributable to other pathogens and (2) children with acute infections at other sites or comorbidities with other diseases. According to the diagnosis and treatment protocol [14], the enrolled children were classified into four clinical types. The methods are shown in Table 1.

**Table 1.** Criteria for four clinical types.

Clinical types	Detailed content
Mild type	Mild clinical symptoms and no pneumonia manifestations on imaging
Common type	Fever, respiratory symptoms, etc., and pneumonia manifestations on imaging
Severe type	Patient requires any of the following: persistent high fever for more than 3 days; presence of tachypnoea (<2 months of age, RR ≥ 60 beats/min; 2 to 12 months of age, RR ≥ 50 beats/min; 1 to 5 years of age, RR ≥ 40 beats/min; >5 years of age, RR ≥ 30 beats/min), except for the effects of fever and crying; resting state, finger oxygen saturatio*n* ≤ 93% on air inhalation; assisted respiration (nasal flapping, tricuspid sign); presence of drowsiness and convulsions; refusal of food or feeding difficulties with signs of dehydration.
Critical type	Patient requires any of the following: respiratory failure and the need for mechanical ventilation; the presence of shock; the combination of other organ failure requiring ICU monitoring treatment

General demographic and epidemiological data included sex, age, vaccination history, close contact or travel history. Clinical symptoms consist of fever, cough, pharyngeal discomfort, fatigue, nasal obstruction, diarrhoea, hypogeusia, hyposmia, dizziness, chest distress, and conjunctival congestion. Children with clinical symptoms were in the symptom-positive group, and children without clinical symptoms were in the symptom-negative group. Axillary temperature was classified as low-grade fever (37.1–38 °C), moderate-grade fever (38–39 °C), high-grade fever (39–41 °C) and ultra-high grade fever (>41 °C). Laboratory tests involve blood cell analysis, T-lymphocyte absolute counts, coagulation function and comprehensive biochemistry.

### CT scanning and evaluation

CT scans were performed using Toshiba and Philips 64-layer spiral CT. CT parameters: 110 kV tube voltage, 100 mA tube current, 5 mm layer thickness, 2.5 mm reconstruction interval and 1.5 mm thin layer. Most patients underwent a high-resolution chest CT on the day of admission or the next day and received one to three follow-up visits within three weeks. The description and scoring of CT images were done independently by two radiologists with clinical experience, and in case of disagreement, a senior physician participated in the joint decision.

Children with at least one positive finding on each CT examination were defined as CT-positive cases. All CT-positive cases' initial and follow-up imaging data were divided into three periods according to the time from symptom onset to the CT scan. CT images were classified into early stage (0–3 days), intermediate stage (3–7 days) and late stage (>7 days). The opacity characteristics include ground-glass opacity (which appears as hazy increased opacity in the lung, with the preservation of bronchial and vascular margins) and consolidation (which appears as a homogeneous increase in pulmonary parenchymal attenuation that obscures the margins of vessels and airway walls) [15]. If the two characteristics appeared simultaneously, they were recorded as GGO and consolidation. If they appeared separately, they were respectively recorded as GGO and consolidation. If there was a small amount of ground-glass opacity around the consolidation or if the consolidation was poorly defined, it was counted as a consolidation, and if there was an independent ground-glass opacity in addition to the consolidation, it was counted as GGO and consolidation. For consolidation, halo signs (appearing as GGO surrounding a nodule or mass) were specifically recorded [15]. Other CT signs (bronchial mucus plug, localized thickening of the interlobular pleura, limited emphysema distal to the lesion, pulmonary fibrosis, pleural effusion and lymph node enlargement) were accurately recorded. Distribution features include central distribution (predominantly in the inner two-thirds of the lung), peripheral distribution (predominantly in the outer third of the lung) and distribution along the bronchial tree. Involved lungs were recorded as unilateral single, unilateral multiple or bilateral multiple. In addition, the number of times each lobe was involved was recorded. The CT score was evaluated by the area involved, with 0–5. Score 0 stands for normal performance (0%), score 1 for minimal (1%–25%), score 2 for mild (26%–50%), score 3 for moderate (51%–75%) and score 4 for severe (76%–100%). The scores of the five lung lobes were summed to yield an overall lung severity score (0–25 points). The CT scores were divided into three groups, grouped by 0–2, 3–5 and 6–9.

### Statistical analysis

All data were analysed with statistical software (SPSS statistical package, version 26; IBM Corporation). The histograms were plotted using OriginPro 2018 C. Scatter plots and ROC curves were produced by GraphPad Prism (Version 9.3.0). The normal distribution of the data was tested using the Shapiro-Wilk test. The data with normal distribution were expressed by $\bar{x}$ ± s, and the independent sample *t*-test compared two groups. The data without normal distribution were expressed by M (P25, P75), and the Mann–Whitney U test compared two groups; The count data were expressed as cases (%), and the comparison was made by the Chi-square test or Fisher's exact test.

Risk factors were assessed between groups using binary logistic regression. The presence or absence of symptoms was used as the dependent variable (1 for the presence of clinical symptoms and 0 for the absence of symptoms), and the independent variables with statistically significant differences between the two groups with and without symptoms were subjected to binary logistic regression analysis. Similarly, the clinical type was used as the dependent variable (1 for common type and 0 for mild type), and the statistically different variables between types were used as independent variables for binary logistic regression analysis. Correlation analysis of CT scores with laboratory findings was performed using Spearman correlation analysis. For some data below the low limit that could not be measured, the data were converted to count data for statistical purposes. *p* < .05 was considered a statistically significant difference.

## Results

### General demographic and epidemiological data of paediatric patients

Fifty-three children were included in the analysis, 25 (47%) were female and 28 (53%) were male, with a mean age of 9.24 ± 5.2 years. All children were not vaccinated. Eighty-nine percent of children had a history of close contact with a COVID-19 person, a history of residence in the community where the case was reported, or a history of contacts in the area where the active case was traced. Family aggregation of infection was common (64%).

### Clinical symptoms of paediatric patients

**Figure 1.**
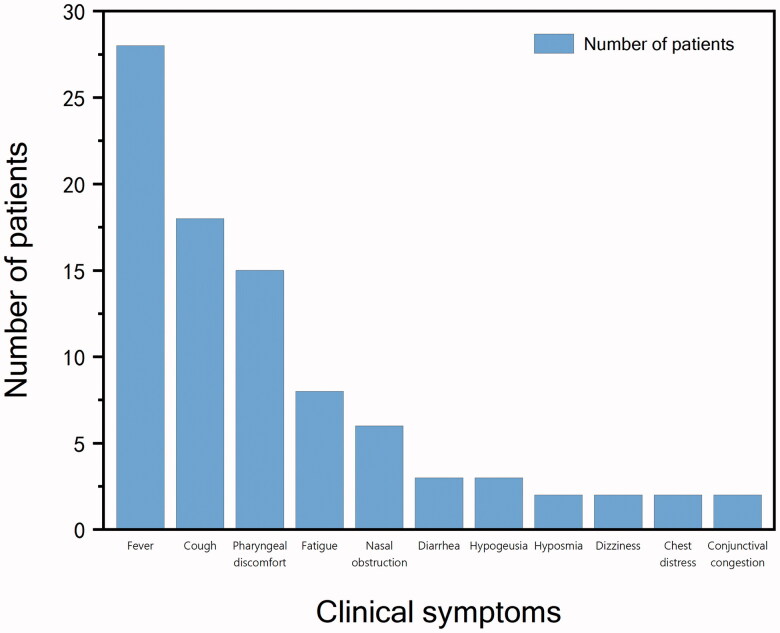
Frequency distribution graph of clinical symptoms.

Of the 53 paediatric patients included 38 cases of mild type and 15 cases of the common type. Nine (17%) were clinically asymptomatic. Among all children, the most common symptoms and frequency were fever 28 (53%), cough 18 (34%) and pharyngeal discomfort 15 (28%). Twenty-four children could be traced to previous peak fever, including 8 (33%) cases of low-grade fever, 14 (58%) cases of moderate-grade fever and 2 (8%) cases of high-grade fever. Cough was classified as dry cough in 15 (83%) cases and cough with sputum in 3 (17%) cases. Pharyngeal discomfort manifested in various ways, such as pharyngeal itching in 5 (33%) cases, dryness of the throat in 2 (13%) cases and pharyngeal pain in 8 (53%) cases. Other relatively rare symptoms included fatigue 8 (15%), nasal congestion 6 (11%), diarrhoea 3 (6%), hypogeusia 3 cases, dizziness 2 (4%), chest discomfort 2 (4%) and conjunctival congestion 2 (4%). The frequency of the above symptoms is shown in Figure 1. A comparison of the frequency of symptoms between the mild and common types is shown in Table 2. The differences between the two types were not statistically significant. The results of the binary logistic regression analysis showed that indicators with differences between groups with and without symptoms did not have a significant effect on the presence or absence of symptoms.

**Table 2.** Clinical feature of different types.

Clinical symptoms	Mild type (*n* = 38)	Common type (*n* = 15)	*p* value
Fever	19 (50%)	9 (60%)	>.05
Cough	11 (29%)	7 (47%)	>.05
Pharyngeal discomfort	10 (26%)	5 (33%)	>.05
Fatigue	7 (18%)	1 (7%)	>.05
Nasal obstruction	5 (13%)	1 (7%)	>.05
Diarrhoea	1 (3%)	2 (13%)	>.05
Hypogeusia	1 (3%)	2 (13%)	>.05
Hyposmia	0 (0%)	2 (13%)	>.05
Dizziness	2 (5%)	0 (0%)	>.05
Chest distress	1 (3%)	1 (7%)	>.05
Conjunctival congestion	2 (5%)	0 (0%)	>.05

The values in parentheses represent the percentage of symptoms present in that clinical subtype.

### Laboratory tests of paediatric patients

**Figure 2.**
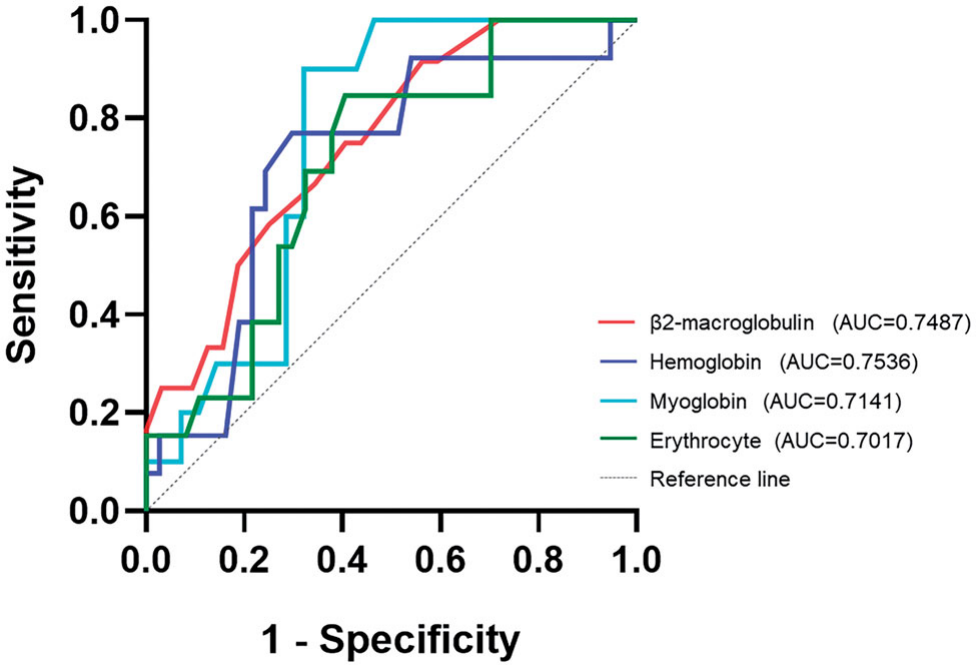
ROC curve.

This study collected patients' blood cell analysis, T-lymphocyte absolute counts, coagulation function and comprehensive biochemistry. The subitems of laboratory tests with a high frequency of abnormalities (trends of laboratory test subitems in children relative to a given reference range) are shown in Table 3. Notably, some of the subitems change both directions. The most common laboratory abnormalities were mean corpuscular volume (68% of patients presented with decreased), lymphocyte percentages (66% of patients presented with abnormalities, in which 64% were elevated and 2% were decreased) and alkaline phosphatase (66% of patients presented with decreased). Some laboratory test results were absent and were omitted in statistics. The frequency of each laboratory item decreased or elevated was compared between the different clinical types. The frequency of decreased haemoglobin and increased percentage of monocytes was statistically different between the two clinical subgroups (*p* = .037 and .033, respectively). In addition, *t*-tests and rank sum tests showing statistically different laboratory tests between mild and common types are shown in Table 4. The ROC curves for the laboratory test subitems in Table 4 are shown in Figure 2, and the areas under the curves were all greater than 0.7. According to the ROC curve analysis, the Jorden indices of the laboratory tests in the Figure 2 were 0.35 (β2-microglobulin), 0.58 (myoglobin), 0.47 (haemoglobin) and 0.44 (erythrocyte), corresponding to the clinical type discrimination thresholds of 1.75 mg/L, 19.20 ng/ml, 133.5 g/L and 4.70 × 10^12^/L. The binary logistic regression analysis results showed that these variables' effect on the clinical types was insignificant.

**Table 3.** Laboratory tests for paediatric patients.

Items	Sub-items	Reference range	Unit	Decrease	Normal	Elevation
Blood analysis	Packed cell volume	(38–50)	%	(22/50) 44%	(28/50) 56%	(0/50) 0%
	Mean corpuscular volume	(83.9–99.1)	fL	(34/50) 68%	(16/50) 32%	(0/50) 0%
	Haemoglobin	(131––172)	g/L	(24/50) 48%	(26/50) 52%	(0/50) 0%
	Mean haemoglobin content	(27.8–33.8)	pg	(24/50) 48%	(26/50) 52%	(0/50) 0%
	Absolute neutrophil count	(2–7)	10^9^/L	(21/50) 42%	(29/50) 58%	(0/50) 0%
	Neutrophil percentages	(40–75)	%	(15/50) 30%	(35/50) 70%	(0/50) 0%
	Eosinophil count	(0.02–0.5)	10^9^/L	(9/50) 18%	(40/50) 80%	(1/50) 2%
	Eosinophil percentage	(0.5–5)	%	(13/50) 26%	(31/50) 62%	(6/50) 12%
	Absolute monocyte count	(0.12–1 )	10^9^/L	(0/50) 0%	(45/50) 90%	(5/50) 10%
	Monocyte percentages	(3–10)	%	(0/50) 0%	(28/50) 56%	(22/50) 44%
	Platelet count	(85–303)	10^9^/L	(0/50) 0%	(41/50) 82%	(9/50) 18%
	Mean platelet volume	(6.5–11)	fL	(0/48) 0%	(31/48) 65%	(17/48) 35%
	High-sensitivity C-reactive protein	(0–3)	mg/L	(0/50) 0%	(43/50) 86%	(7/50) 14%
	IL-6	(0–6)	pg/ml	(0/46) 0%	(29/46) 63%	(17/46) 37%
	Lymphocyte absolute value	(0.8–4)	10^9^/L	(1/50) 2%	(47/50) 94%	(2/50) 4%
	Lymphocyte percentages	(20–40)	%	(1/50) 2%	(17/50) 34%	(32/50) 64%
T-lymphocyte absolute counts	CD4 + T-cells	(414–1123)	A/ml	(9/44) 21%	(31/44) 70%	(4/44) 9%
	CD4+/CD45+	(30–46)	%	(11/44) 25%	(33/44) 75%	(0/44) 0%
	CD8 + T-cells	(238–874)	A/ml	(4/44) 9%	(33/44) 75%	(7/44) 16%
	CD8+/CD45+	(19.2–33.6)	%	(6/44) 14%	(29/44) 66%	(9/44) 21%
Coagulation function	Fibrinogen	(2–4)	g/L	(13/49) 27%	(36/49) 73%	(0/49) 0%
	Thrombin time	(10–14)	s	(7/49) 14%	(40/49) 82%	(2/49) 4%
	D-dimer	(0–0.55)	mg/L	(0/49) 0%	(43/49) 88%	(6/49) 12%
	Fibrinogen degradation products	( 0–5)	ug/ml	(0/49) 0%	(33/49) 67%	(16/49) 33%
Comprehensive biochemistry	HDL cholesterol	(1.04–1.9)	mmol/L	(13/41) 32%	(27/41) 66%	(1/41) 2%
	Globulin	(20–30)	g/L	(2/51) 4%	(35/51) 69%	(14/51) 28%
	Alkaline phosphatase	(40–129)	IU/L	(33/50) 66%	(17/50) 34%	(0/51) 0%
	Lactate dehydrogenase	(109–245)	IU/L	(30/50) 60%	(20/50) 40%	(0/51) 0%
	α-hydroxybutyrate dehydrogenase	(72–182)	IU/L	(20/43) 47%	(23/43) 53%	(0/43) 0%
	Serum sodium	(136–145)	mmol/L	(11/51) 22%	(40/51) 78%	(0/51) 0%
	Chloride sodium	(96–106)	mmol/L	(0/51) 0%	(37/51) 73%	(14/51) 27%

**Table 4.** Comparison of laboratory tests in mild and common type.

Items	Mild type	Common type	Test statistic	*p* value
β2-microglobulin (x ± s, mg/L)	1.79 ± 0.56	2.31 ± 0.53	−2.78^a^	.01
Myoglobin [M (P25,P75), ng/ml]	17.4 (14.6, 22.4)	21.3 (19.6, 25.6)	−2.35^b^	.02
Haemoglobin [M (P25,P75), g/L]	129.0 (123.5, 135.0)	138.0 (130.5, 142.0)	−2.28^b^	.02
Erythrocyte [M (P25,P75), 10^12^/L]	4.7 (4.5, 4.9)	4.9 (4.7, 5.1)	−2.15^b^	.03

^a^is the Z-value, ^b^is the *t*-value.

### The features of high-resolution chest CT

Fifty-one of the 53 paediatric patients underwent chest CT examinations. The CT imaging features of 15 children were abnormal, with 9 children showing positive findings at the initial CT examination and 6 children showing abnormalities at a subsequent follow-up visit.

**Figure 3.**
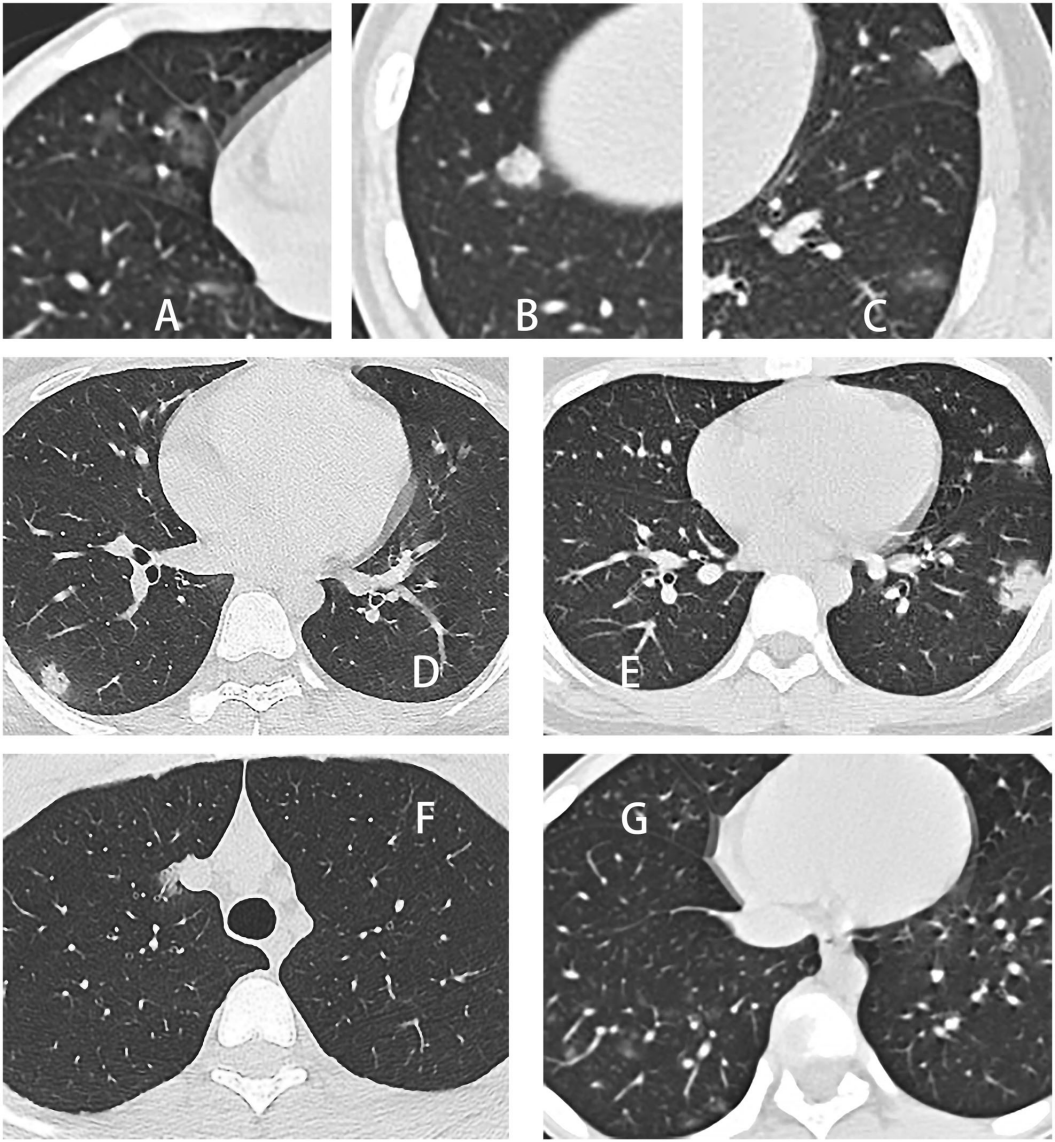
Examples of opacity characteristics and distribution characteristics. (A) Ground-glass opacity in the middle lobe of the right lung. (B) Consolidation of the lower lobe of the right lung. (C) Multiple lesions in the left lung were recorded as GGO and consolidation. (D) A single lesion in the lower lobe of the right lung was recorded as a peripheral distribution. (E) Multiple lesions in the left lung were recorded with a peripheral distribution. (F) A single lesion in the right upper lung was recorded as a central distribution. (G) Multiple lesions in the right lung, mainly located in the peripheral zone of the lower lung, were documented to be distributed along with the bronchial bundles.

**Figure 4.**
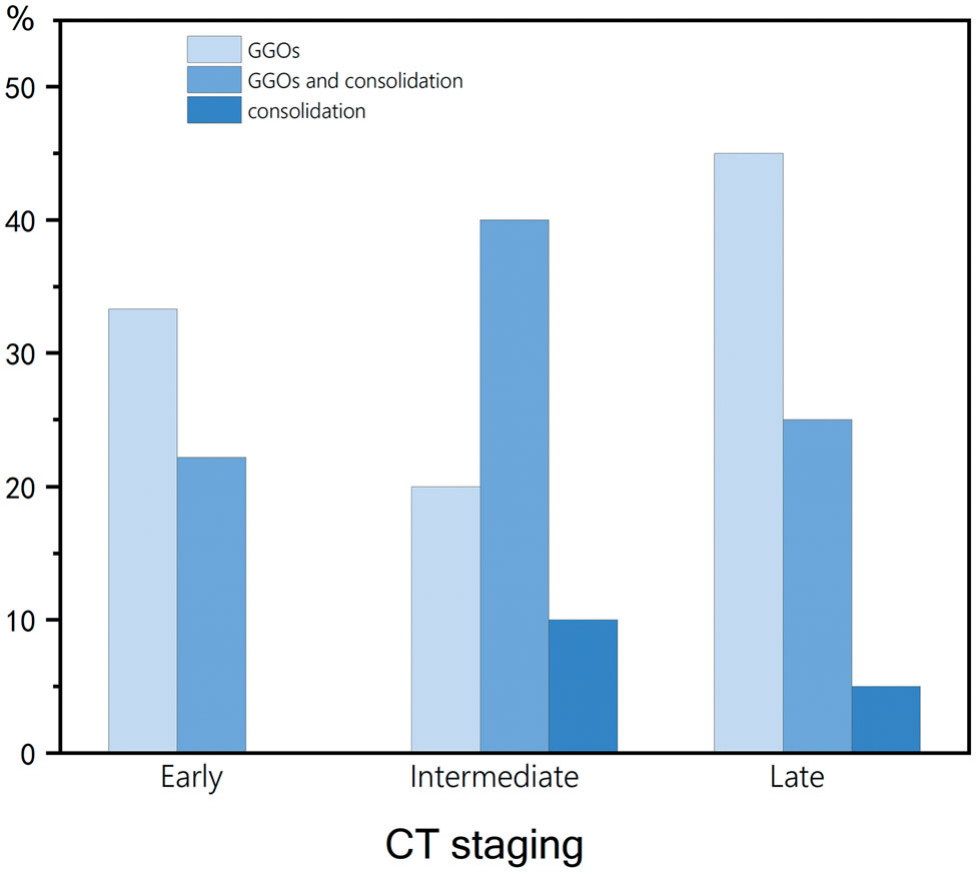
Frequencies of opacity characteristics. The values in parentheses represent the percentage of CT scores within CT stages.

There were 9 cases of CT images in the early stage (1.9 ± 0.9 days), 10 cases in the intermediate stage (4.9 ± 1.0 days) and 20 cases in the late stage (13.5 ± 4.7 days). The different opacity characteristics are shown in Figure 3. In the early stage, three cases (33%) showed GGO, one case (11%) showed consolidation and one case (11%) showed GGO and consolidation. In the intermediate stage, two cases (20%) showed GGO, three cases (30%) showed consolidation and two cases (20%) showed GGO and consolidation. In the late stage, eight cases (40%) showed GGO, three cases (15%) showed consolidation and four cases (20%) showed GGO and consolidation. Ten of 14 (71%) consolidations were accompanied by halo signs. The frequencies of opacity characteristics are shown in Figure 4. Some CT signs, such as bronchial mucus plugs (one case), localized thickening of the interlobular pleura (one case), limited emphysema distal to the lesion (two cases), and pulmonary fibrosis (one case), were seen in fewer children. CT of the chest did not show pleural effusion or lymph node enlargement.

**Figure 5.**
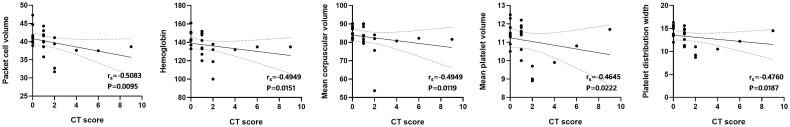
Correlation of CT scores with laboratory tests.

The different distribution characteristics are shown in Figure 3. Twenty-one cases showed predominantly peripheral distribution on CT images. Extended bronchial bundle distribution (three cases) and central lung band distribution (one case) were other rare distributions. Four cases showed multiple unilateral lesions, 11 cases showed single unilateral lesions and 12 cases showed multiple bilateral lesions. All five anatomical lung lobes were involved, in descending order of frequency of involvement: right lower lung (19 cases), left lower lung (12 cases), left upper lung (11 cases), right upper lung (8 cases) and right middle lung (8 cases). Ten cases showed predominantly lower lung, and among these, eight cases showed both lower lung and peripheral distribution. A summary organized by CT score groups is shown in Table 5. Among the three CT score groups, a CT score of 0–2 accounted for the highest percentage, and the number in the CT score groups decreased as the CT score increased. The mean CT scores of the early, intermediate and late stages did not show much difference, and a relatively flat trend could be seen. The CT scores in the intermediate stage were slightly higher than those in the early and late stages. Correlation analysis of CT scores with laboratory tests showed that some of the laboratory test subitems had a moderate correlation with CT scores, as shown in Figure 5.

**Table 5.** CT scores.

CT score	Early (*n* = 9)	Intermediate (*n* = 10)	Late (*n* = 20)	Total (*n* = 39)
0–2	8 (89)%	7 (70%)	16 (80%)	31 (80%)
3–5	0 (0%)	2 (20%)	4 (20%)	6 (15%)
6–9	1 (11%)	1 (10%)	0 (0%)	2 (5%)
Average score	1.3	2.0	1.6	1.6

The values in parentheses represent the percentage of CT scores within CT stages.

## Discussion

In this study, most paediatric patients had a history of close contact with confirmed COVID-19 patients, and 64% of children became ill due to family members' infection and were not index cases. Children were often not the first infections in family aggregated cases, consistent with the trend reported in a meta-analysis addressing the role of children in transmission in new coronavirus pneumonia [16]. The role of children in SARS-CoV-2 transmission is controversial, with some studies suggesting that children may play a key role in transmission, possessing similar viral loads to adults [17–18]. In contrast, other studies have found little evidence of transmission in children [19]. In the case of coronaviruses, paediatric infection is milder [20–21]. The lower incidence of clinical symptoms raises concerns that children may be an important, undetected source of transmission of SARS-CoV-2. Therefore, whether children should be at a priority level for vaccination and community management is a point that needs to be further explored in the future.

Seventeen percent of the children showed no clinical symptoms during the disease. Fever, dry cough and pharyngeal discomfort were the most common manifestations among the remaining children. Paediatric patients had a mild clinical presentation and no obvious specific symptoms. These results are consistent with other paediatric and adult studies [22–25]. Of concern is the small number of nonrespiratory symptoms, such as diarrhoea, loss of smell and taste, which should not be ignored when these non-specific and rare symptoms are present, especially as a single symptom. In such cases, a history of close contact is critical. Two clinical subtypes were involved in this study, and the difference in the frequency of symptoms between the two was not statistically significant. This may be because the patients included in this study had a milder disease, and the two could not be distinguished symptomatically. The ROC curves showed no influential factors significantly contributing to the presence or absence of symptoms in the patients, which also indicates that age, gender and laboratory tests could not influence whether the children showed clinical symptoms or not.

The generally milder symptom profile of paediatric patients has different explanations. (1) SARS-CoV-2 entering the human body will infect endothelial cells. The activation of coagulation pathways and the formation of microthrombi due to endothelial damage are essential in the pathogenesis of COVID-19 [26–27]. Compared with adults, children have healthier endothelial cells and lack vascular risk factors and a different coagulation system, making them less prone to abnormal coagulation [28]. In this study, a small number of coagulation abnormalities were reported and to a lesser extent. (2) There are differences in childhood immune responses. Different studies differ regarding the differences in immune response in children with coronavirus disease 2019. Some studies suggest that stronger innate immunity will clear the virus in children more effectively. In addition, the weaker adaptive immune response in children allows for a milder inflammatory response [29]. In addition to a protective effect, it has also been suggested that a more robust innate immune response may be both protective and may also worsen the cytokine storm [30–31]. Most of the inflammatory indicators in this study were within the normal range, which seems to support the protective role of the immune system in children. (3) T-cell immunity in children is different. In SARS-CoV-2, the role of cross-reactive T-cells remains unclear. It has been shown that low-activity T-cells, usually high in the elderly, negatively affect the T-cell response to SARS-CoV-2 [32]. This could also explain why the clinical presentation is more severe in elderly patients with COVID-19. On the other hand, paediatric patients with COVID-19 had weaker T-cell responses to the spike protein [29]. Of note are the T-cell alterations observed in this study. Sixty-four percent of the children showed an increase in the percentage of lymphocytes. The relatively apparent trend in further lymphocyte sorting counts was a decrease in CD4+ T-cell count and CD4+/CD45+ ratio and an increase in CD8+ T-cell count and CD8+/CD45+ ratio. Regarding the opposite trends of these two T-cells, an interrelationship has been suggested, with CD4+ regulatory T-cells producing IL-10 to promote memory CD8+ T-cell maturation during infection regression [33]. However, further immunological studies are needed to confirm the role of both during recovery from SARS-CoV-2 infection. CD4+ T-cell reduction is common in adults with severe and moderate COVID-19 but was only seen in a small number of children in this study [34], consistent with the milder course of the disease.

Elevated alkaline phosphatase, lactate dehydrogenase and alpha hydroxybutyrate dehydrogenase may indicate liver damage. Previous studies have also found elevated liver enzymes in paediatric patients [35]. Decreased erythrocyte volume, haemoglobin and mean haemoglobin content suggest anaemia. Different studies have had different results regarding anaemia changes in patients. Some studies have shown that decreased haemoglobin levels in COVID-19 patients correlate with the severity of the disease [36]. However, in contrast, some studies have reported similar total haemoglobin levels in patients with COVID-19 compared with those without COVID-19 infection [37], and some studies have even reported slightly higher haemoglobin in patients with COVID-19 than in normal controls [38]. Therefore, further studies are still needed to clarify the relationship between anaemia changes and the disease in children.

The positive rate was only 29% in CT examinations and was even lower at 18% in the initial CT examination. This suggests a lower rate of CT positivity in paediatric patients, which appears to be lower than previous studies in adults or children [9,39]. The current understanding of the infectiousness of the SARS-CoV-2 B.1.617.2 (Delta) variant is that it is more infectious than prototypic SARS-CoV-2 [40–41]. Similarly, the literature has reported that it is also more pathogenic [42]. However, in the CT presentation of the paediatric samples included in this study, the imaging changes were generally mild. There are several hypotheses regarding this positive rate: (1) The B.1.617.2 (Delta) variant causes less lung damage in children, and the child protective factors mentioned above remain effective in this variant infection. (2) The small number of patients included in this study and the fact that they were all mild and common type and not representative. Whether the high infectivity and pathogenicity of the B.1.617.2 (Delta) variant in mice can be expanded in the paediatric population still needs to be confirmed by future clinical cohort studies. CT only provides some screening significance and cannot be relied upon to exclude the possibility of disease.

In this study, the major opacity characteristics of COVID-19 were GGO and consolidation, which is consistent with previous prototypic SARS-CoV-2 studies [39,43]. In animal models, focal inflammatory cell infiltrates were observed around the bronchi/bronchioles one day after infection, with haemorrhage or congestion present at three days. Subsequently, crushed nuclear fragments were observed, indicating alveolar pneumocyte damage and the area of inflammatory cell infiltration expanded over time [42]. These different CT presentations are associated with complex pathological changes in the lungs. Microscopically, plasma is seen in the alveolar cavity [14], which may be the pathological basis of GGO, and fibrinous exudate is associated with further consolidation transformation of GGO. Consolidation areas mainly showed diffuse alveolar damage and exudative alveolitis. Alveolar septa are congested and oedematous with mononuclear and lymphocytic infiltration (Diagnosis and Treatment Protocols of Coronavirus Disease 2019. 2021). In this study, thickening of the lobular septa was not observed. A few alveoli were hyperinflated, with alveolar septum rupture or cyst formation. In a few patients, restrictive emphysema was found distal to the lesion. The epithelium of the bronchial mucosa in the lungs was detached, and exudate and mucus were visible in the lumen. Accordingly, a small amount of small bronchial and fine bronchial mucus plug formation was observed in this study. The most frequently involved lung region was the right lung, and the most frequently involved lobe was the right lower lung, followed by the left lower lung, which is consistent with previous reports [43].

The limitations of this study are first the small number of cases included in the study and the even smaller sample of imaging data with positive CT findings, which requires a large sample of data for further study. Second, some patients included in this study were already treated in other hospitals. The effect of the treatment regimen prior to hospital admission on chest CT examinations could not be estimated. Third, because the study was retrospective, the CT data collected from the patients included did not follow a strict schedule. This resulted in not every patient having imaging data collected for all three periods, detrimental to observing treatment changes and prognosis. Further standardization of data collection and studies is still needed.

In conclusion, the clinical presentation of paediatric patients was mild and influenced by numerous factors. The most common symptoms were fever, cough and pharyngeal discomfort, and the clinical diagnosis needs to be integrated with the epidemiological history. Laboratory findings were not significantly specific, and the extent of change was milder than in adults. The CT positivity rate was also low, mainly manifesting as GGO and consolidation. Pulmonary involvement was mild, and the CT score was low and varied with disease progression. CT of the chest can indicate the course of the disease and pathological changes, thus guiding treatment.

## Data Availability

Raw data were generated at [Nanjing Second Hospital (Tangshan Branch)]. Derived data supporting the findings of this study are available from the corresponding author [H] on request.

## References

[1] Gorbalenya AE, Baker SC, Baric RS, et al. The species severe acute respiratory syndrome-related coronavirus: classifying 2019-nCoV and naming it SARS-Cov-2. Nat Microbiol. 2020;5(4):536–544. 32123347 10.1038/s41564-020-0695-z PMC7095448

[2] Starr TN, Greaney AJ, Dingens AS, et al. Complete map of SARS-CoV-2 RBD mutations that escape the monoclonal antibody LY-CoV555 and its cocktail with LY-CoV016. Cell Rep Med. 2021;2(4):100255. 33842902 10.1016/j.xcrm.2021.100255 PMC8020059

[3] Joshi N, Tyagi A, Nigam S. Molecular level dissection of critical spike mutations in SARS-Cov-2 variants of concern (VOSs): a simplified review. Chemistryselect. 2021;6(31):7981–7998. 34541298 10.1002/slct.202102074 PMC8441688

[4] Tareq AM, Emran TB, Dhama K, et al. Impact of SARS-CoV-2 Delta variant (B.1.617.2) in surging second wave of covid-19 and efficacy of vaccines in tackling the ongoing pandemic. Hum Vaccines Immunother. 2021; 17: 4126–4127. 10.1080/21645515.2021.1963601 PMC8425453 34473593

[5] Jiangsu Commission of Health. http://wjw.jiangsu.gov.cn/art/2021/7/21/art_7290_9893771.html. (Accessed July 28, 2022)

[6] Zavascki AP, Falci DR. Clinical characteristics of COVID-19 in China. N Engl J Med. 2020;382(19):1859–1859. 32220202 10.1056/NEJMc2005203

[7] Matthay MA, Zemans RL, Zimmerman GA, et al. Acute respiratory distress syndrome. Nat Rev Dis Primers. 2019;5(1):18. 30872586 10.1038/s41572-019-0069-0 PMC6709677

[8] Ludvigsson JF. Systematic review of COVID-19 in children shows milder cases and a better prognosis than adults. Acta Paediatr. 2020;109(6):1088–1095. 32202343 10.1111/apa.15270 PMC7228328

[9] Patel NA. Pediatric COVID-19: systematic review of the literature. Am J Otolaryngol. 2020;41(5):102573. 32531620 10.1016/j.amjoto.2020.102573 PMC7833675

[10] Liguoro I, Pilotto C, Bonanni M, et al. SARS-CoV-2 infection in children and newborns: a systematic review. Eur J Pediatr. 2020;179(7):1029–1046. 32424745 10.1007/s00431-020-03684-7 PMC7234446

[11] Lu XX, Zhang LQ, Du H, Chinese Pediatric Novel Coronavirus Study Team, et al. SARS-Cov-2 infection in children. N Engl J Med. 2020;382(17):1663–1665. 32187458 10.1056/NEJMc2005073 PMC7121177

[12] Henry BM, Lippi G, Plebani M. Laboratory abnormalities in children with novel coronavirus disease 2019. Clin Chem Lab Med. 2020;58(7):1135–1138. 32172227 10.1515/cclm-2020-0272

[13] Ebrahimpour L, Marashi M, Zamanian H, et al. Computed tomography findings in 3,557 COVID-19 infected children: a systematic review. Quant Imaging Med Surg. 2021;11(11):4644–4660. +. 34737930 10.21037/qims-20-1410 PMC8511729

[14] National Health Commission of the People's Republic of China: Diagnosis and Treatment Protocols of Coronavirus Disease 2019 (Trial Version 8 Revised). 2021. http://www.nhc.gov.cn/yzygj/s7653p/202104/7de0b3837c8b4606a0594aeb0105232b.shtml. (Accessed November 10, 2021).

[15] Hansell DM, Bankier AA, MacMahon H, et al. Fleischner society: glossary of terms for thoracic imaging. Radiology. 2008;246(3):697–722. 18195376 10.1148/radiol.2462070712

[16] Zhu Y, Bloxham CJ, Hulme KD, et al. A meta-analysis on the role of children in severe acute respiratory syndrome coronavirus 2 in household transmission clusters. Clin Infect Dis. 2021;72(12):E1146–E1153. 33283240 10.1093/cid/ciaa1825 PMC7799195

[17] Yonker LM, Neilan AM, Bartsch Y, et al. Pediatric severe acute respiratory syndrome coronavirus 2 (SARS-CoV-2): clinical presentation, infectivity, and immune responses. J Pediatr. 2020;227:45–52.e45. 32827525 10.1016/j.jpeds.2020.08.037 PMC7438214

[18] Jones TC, Biele G, Mühlemann B, et al. Estimating infectiousness throughout SARS-CoV-2 infection course. Science, 2021, 373(6551):eabi5273. 10.1126/science.abi5273 PMC9267347 34035154

[19] Lee B, Raszka WVJP. COVID-19 transmission and children: the child is not to blame. Pediatrics. 2020;146(2):e2020004879 32457212 10.1542/peds.2020-004879

[20] Szablewski CM, Chang KT, Brown MM, et al. SARS-CoV-2 transmission and infection among attendees of an overnight camp—Georgia. MMWR Morb Mortal Wkly Rep. 2020;69(31):1023–1025. 32759921 10.15585/mmwr.mm6931e1 PMC7454898

[21] Weycker D, Edelsberg J, Halloran ME, et al. Population-wide benefits of routine vaccination of children against influenza. Vaccine. 2005;23(10):1284–1293. 15652671 10.1016/j.vaccine.2004.08.044

[22] Chan JF-W, Yuan S, Kok K-H, et al. A familial cluster of pneumonia associated with the 2019 novel coronavirus indicating person-to-person transmission: a study of a family cluster. Lancet. 2020;395(10223):514–523. 31986261 10.1016/S0140-6736(20)30154-9 PMC7159286

[23] Dong Y, Mo X, Hu Y, et al. Epidemiology of COVID-19 among children in China. Pediatrics. 2020;145(6):e20200702 32179660 10.1542/peds.2020-0702

[24] Duan Y-N, Zhu Y-Q, Tang L-L, et al. CT features of novel coronavirus pneumonia (COVID-19) in children. Eur Radiol. 2020;30(8):4427–4433. 32291501 10.1007/s00330-020-06860-3 PMC7156230

[25] Zimmermann P, Curtis N. Coronavirus infections in children including COVID-19 an overview of the epidemiology, clinical features, diagnosis, treatment and prevention options in children. Pediatr Infect Dis J. 2020;39(5):355–368. 32310621 10.1097/INF.0000000000002660 PMC7158880

[26] Tang N, Li D, Wang X, et al. Abnormal coagulation parameters are associated with poor prognosis in patients with novel coronavirus pneumonia. J Thromb Haemost. 2020;18(4):844–847. 32073213 10.1111/jth.14768 PMC7166509

[27] Varga Z, Flammer AJ, Steiger P, et al. Endothelial cell infection and endotheliitis in COVID-19. Lancet. 2020;395(10234):1417–1418. 32325026 10.1016/S0140-6736(20)30937-5 PMC7172722

[28] Ignjatovic V, Mertyn E, Monagle P. The coagulation system in children: developmental and pathophysiological considerations. Semin Thromb Hemost. 2011;37(7):723–729. 22187394 10.1055/s-0031-1297162

[29] Pierce CA, Preston-Hurlburt P, Dai Y, et al. Immune responses to SARS-CoV-2 infection in hospitalized pediatric and adult patients. Sci Transl Med. 2020;12(564):eabd5487 32958614 10.1126/scitranslmed.abd5487 PMC7658796

[30] Jose RJ, Manuel A. COVID-19 cytokine storm: the interplay between inflammation and coagulation. Lancet Respir Med. 2020;8(6):e46–e47. 32353251 10.1016/S2213-2600(20)30216-2 PMC7185942

[31] Mehta P, McAuley DF, Brown M, HLH Across Speciality Collaboration, UK, et al. COVID-19: consider cytokine storm syndromes and immunosuppression. Lancet. 2020;395(10229):1033–1034. 32192578 10.1016/S0140-6736(20)30628-0 PMC7270045

[32] Bacher P, Rosati E, Esser D, et al. Low-avidity CD4+ T cell responses to SARS-CoV-2 in unexposed individuals and humans with severe COVID-19. Immunity. 2020;53(6): 1258–1271. e5. 10.1016/j.immuni.2020.11.016 PMC7689350 33296686

[33] Laidlaw BJ, Cui W, Amezquita RA, et al. Production of Il-10 by Cd4+ regulatory T cells during the resolution of infection promotes the maturation of memory Cd8+ T cells. Nat Immunol. 2015;16(8):871–879. 26147684 10.1038/ni.3224 PMC4713030

[34] QinC Z. Dysregulation of immune response in patients with COVID-19 in Wuhan, China. Clin Infect Dis. 2020;71:762–768. 32161940 10.1093/cid/ciaa248 PMC7108125

[35] de Souza TH, Nadal JA, Nogueira RJ, et al. Clinical manifestations of children with COVID‐19: a systematic review. Pediatr Pulmonol. 2020;55(8):1892–1899. 32492251 10.1002/ppul.24885 PMC7300659

[36] Karimi Shahri M, Niazkar HR, Rad F. COVID‐19 and hematology findings based on the current evidences: a puzzle with many missing pieces. Int J Lab Hematol. 2021;43(2):160–168. 33264492 10.1111/ijlh.13412 PMC7753300

[37] DeMartino AW, Rose JJ, Amdahl MB, et al. No evidence of hemoglobin damage by SARS-CoV-2 infection. Haematologica. 2020;105(12):2769–2773. 33054129 10.3324/haematol.2020.264267 PMC7716349

[38] Pascual-Guàrdia S, Ferrer A, Díaz Ó, et al. Absence of relevant clinical effects of SARS-CoV-2 on the affinity of hemoglobin for O_2_ in patients with COVID-19. Arch Bronconeumol. 2021;57:757–763 34720331 10.1016/j.arbr.2021.10.010 PMC8536567

[39] Liu K-C, Xu P, Lv W-F, et al. CT manifestations of coronavirus disease-2019: a retrospective analysis of 73 cases by disease severity. Eur J Radiol. 2020;126:108941. 32193037 10.1016/j.ejrad.2020.108941 PMC7118536

[40] Kumar V, Singh J, Hasnain SE, et al. Possible link between higher transmissibility of alpha, kappa and Delta variants of SARS-CoV-2 and increased structural stability of its spike protein and Hace2 affinity. IJMS. 2021;22(17):9131. 34502041 10.3390/ijms22179131 PMC8431609

[41] Arora P, Sidarovich A, Krüger N, et al. B. 1.617. 2 enters and fuses lung cells with increased efficiency and evades antibodies induced by infection and vaccination. Cell Rep. 2021;37(2):109825. 34614392 10.1016/j.celrep.2021.109825 PMC8487035

[42] Saito A, Irie T, Suzuki R, The Genotype to Phenotype Japan (G2P-Japan) Consortium, et al. Enhanced fusogenicity and pathogenicity of SARS-CoV-2 Delta P681r mutation. Nature. 2022;602(7896):300–310. 34823256 10.1038/s41586-021-04266-9 PMC8828475

[43] Xia W, Shao J, Guo Y, et al. Clinical and Ct features in pediatric patients with COVID‐19 infection: different points from adults. Pediatr Pulmonol. 2020;55(5):1169–1174. 32134205 10.1002/ppul.24718 PMC7168071

